# Exploring molecular mechanisms of exercise on metabolic syndrome: a bibliometric and visualization study using CiteSpace

**DOI:** 10.3389/fendo.2024.1408466

**Published:** 2024-09-03

**Authors:** Kang Wan, Yue Jin, Ruobing Fan, Qizi Xu, Xiaoshi Li, Hongmei Yan, Ru Wang

**Affiliations:** ^1^ School of Exercise and Health, Shanghai University of Sport, Shanghai, China; ^2^ Physical Education College, Henan Sport University, Zhengzhou, China; ^3^ Department of Endocrinology and Metabolism, Zhongshan Hospital, Fudan University, Shanghai, China; ^4^ Department of Endocrinology and Metabolism, Wusong Branch of Zhongshan Hospital, Fudan University, Shanghai, China

**Keywords:** exercise, metabolic syndrome, molecular mechanisms, visualization analysis, CiteSpace

## Abstract

**Objective:**

To investigate the molecular mechanisms through which exercise influences metabolic syndrome (MS) and identify key research trends and collaborative networks using bibliometric and visualization techniques.

**Methods:**

We conducted a systematic literature search using the Web of Science Core Collection for articles published from 2014 to 2023. Using CiteSpace, we performed a bibliometric analysis of 562 eligible papers, generating visual knowledge maps to identify prevailing patterns, popular subjects, and emerging trends in the literature.

**Results:**

The study reveals that exercise mitigates MS by reversing high-fat diet-induced abdominal obesity, reducing lipid accumulation and inflammation, enhancing insulin sensitivity, and improving cardiovascular function. Key molecular pathways include PPAR-γ/CPT-1/MCAD signaling, AMPK activation, and nitric oxide production. The USA leads in research output, with significant contributions from American institutions. Collaboration among researchers is limited, highlighting the need for more extensive and high-quality research initiatives.

**Conclusions:**

Regular, moderate-to-high-intensity exercise is crucial for managing MS. Exercise activates beneficial molecular pathways, improving metabolic health and cardiovascular function. Future research should focus on expanding collaborations and exploring novel molecular targets to enhance the therapeutic potential of exercise in metabolic syndrome management.

## 1 Introduction

Metabolic syndrome (MS) is a complex set of metabolic disorders that includes central obesity, insulin resistance, hypertriglyceridemia, hypercholesterolemia, hypertension, and reduced high-density lipoprotein cholesterol concentrations (1–4). Metabolic syndrome (MS) is defined in many ways by different specialists, and these classifications have undergone changes throughout time. The criteria for multiple sclerosis (MS) were initially defined by the World Health Organization (WHO) in 1998. The criteria encompassed central obesity and several metabolic abnormalities, with diabetes or insulin resistance being a prominent underlying risk factor (5). Insulin resistance is a key diagnostic indicator for multiple sclerosis (MS), together with the presence of two or more other risk factors such as obesity, hyperglycemia, hypertension, and high blood triglycerides (6, 7). Subsequently, the International Diabetes Federation (IDF) added abdominal obesity as one of the five essential criteria for diagnosis, eliminating the need for insulin resistance (8). Insulin resistance was not considered a priority in the 2001 guidelines established by the Adult Treatment Panel III (ATP III) of the National Cholesterol Education Program (9). In 2009, the International Diabetes Federation (IDF), American Heart Association (AHA), and International Atherosclerosis Society (IAS) issued a joint statement that introduced the initial standardized definition of metabolic syndrome (MS). According to this definition, three metabolic abnormalities can confirm the diagnosis of MS, and the presence of the five major components of MS is not mandatory (10, 11). We remain uncertain about the pathogenic mechanisms and etiological factors of metabolic syndrome (MS), as any of its several components could be the cause. An intricate array of underlying factors influences each component, and the ongoing exploration of novel mechanisms is constant. Specific factors contributing to many health conditions include genetics, insulin resistance, obesity, lifestyle choices, sleep disturbances, inflammatory responses, early childhood and neonatal settings, and disruptions in circadian rhythms (12–14). Among these, lifestyle factors such as prolonged sedentary behavior, lack of regular physical activity, and unhealthy dietary habits are important causes of MS (15–17).

Exercise and calorie restriction are essential management strategies for metabolic syndrome, as clinical guidelines recommend comprehensive lifestyle modifications as the first-line treatment (18). Exercise training is crucial to promoting cardiovascular health, as it can improve specific target parameters such as glucose control, lipid status, and physical fitness (19, 20). Additionally, numerous studies have demonstrated that higher levels of cardiorespiratory fitness reduce the risk of developing metabolic syndrome (21, 22). The characteristics of exercise, including frequency, intensity, type, and duration, differentially influence physiological outcomes. Endurance exercise enhances the body’s oxygen uptake capacity and improves vascular function, while resistance training with gradual loading stimulates skeletal muscle growth. Consequently, research has shown that therapies combining endurance and resistance training are more effective in improving glycemic control, anti-inflammatory responses, and body composition (23–28). Despite the widespread recognition of exercise’s positive effects on metabolic syndrome, the specific molecular pathways still remain unclear, especially when it comes to how exercise impacts abdominal obesity, abnormal lipid metabolism, inflammation, insulin sensitivity, and cardiovascular function (4, 19, 29). More in-depth studies are needed to uncover the signaling pathways and effector molecules involved in exercise.

CiteSpace, a sophisticated Java-based software for scientometric and bibliometric analysis, was employed in this study to explore the molecular mechanisms by which exercise influences metabolic syndrome. By utilizing advanced techniques such as cluster analysis, co-occurrence analysis, and metrology, we identified research frontiers and hotspots within the literature (30). This innovative approach enabled us to compile and visually represent a vast array of articles, shedding light on the primary sources of knowledge, trending subjects, and the historical evolution of this research field. Our comprehensive analysis provides new insights into the complex molecular effects of physical activity on metabolic disorders, highlighting pivotal findings and emerging areas of study. The study’s findings offer valuable guidance for future research directions and emphasize crucial considerations for researchers and policymakers in integrating exercise or physical activity into strategies for the prevention and management of metabolic syndrome.

## 2 Methods

### 2.1 Retrieval strategy

We retrieved articles from the Web of Science Core Collection Science Citation Index Expanded (SCI-Expanded) for the period between January 1, 2014, and December 31, 2023, using the following search terms: exercise, metabolic syndrome, and molecular mechanism. **Table 1** shows the detailed search strategy.

**Table 1:** Retrieval strategy.

Set	Search query
#1	TS=(Exercise OR Exercises OR Physical Activity OR Activities, Physical OR Activity, Physical OR Physical Activities OR Exercise, Physical OR Exercises, Physical OR Physical Exercise OR Physical Exercises OR Acute Exercise OR Acute Exercises OR Exercise, Acute OR Exercises, Acute OR Exercise, Isometric OR Exercises, Isometric OR Isometric Exercises OR Isometric Exercise OR Exercise, Aerobic OR Aerobic Exercise OR Aerobic Exercises OR Exercises, Aerobic OR Exercise Training OR Exercise Trainings OR Training, Exercise OR Trainings, Exercise)
#2	TS= (Metabolic syndrome OR Metabolic Syndromes OR Syndrome, Metabolic OR Syndromes, Metabolic OR Metabolic Syndrome X OR Insulin Resistance Syndrome X OR Syndrome X, Metabolic OR Syndrome X, Insulin Resistance OR Syndrome X, Insulin Resistance OR Syndrome, Metabolic X OR X Syndrome, Metabolic OR Dysmetabolic Syndrome X OR Syndrome X, Dysmetabolic OR Reaven Syndrome X OR Syndrome X, Reaven OR Metabolic Cardiovascular Syndrome OR Cardiovascular Syndrome, Metabolic OR Cardiovascular Syndromes, Metabolic OR Syndrome, Metabolic Cardiovascular OR Cardiometabolic Syndrome OR Cardiometabolic Syndromes OR Cardiometabolic Syndromes OR Syndromes, Cardiometabolic)
#3	(#1) AND (#2)
#4	[TS= (molecular mechanism)] AND (#3)

### 2.2 Inclusion and exclusion criteria

**Figure 1:**
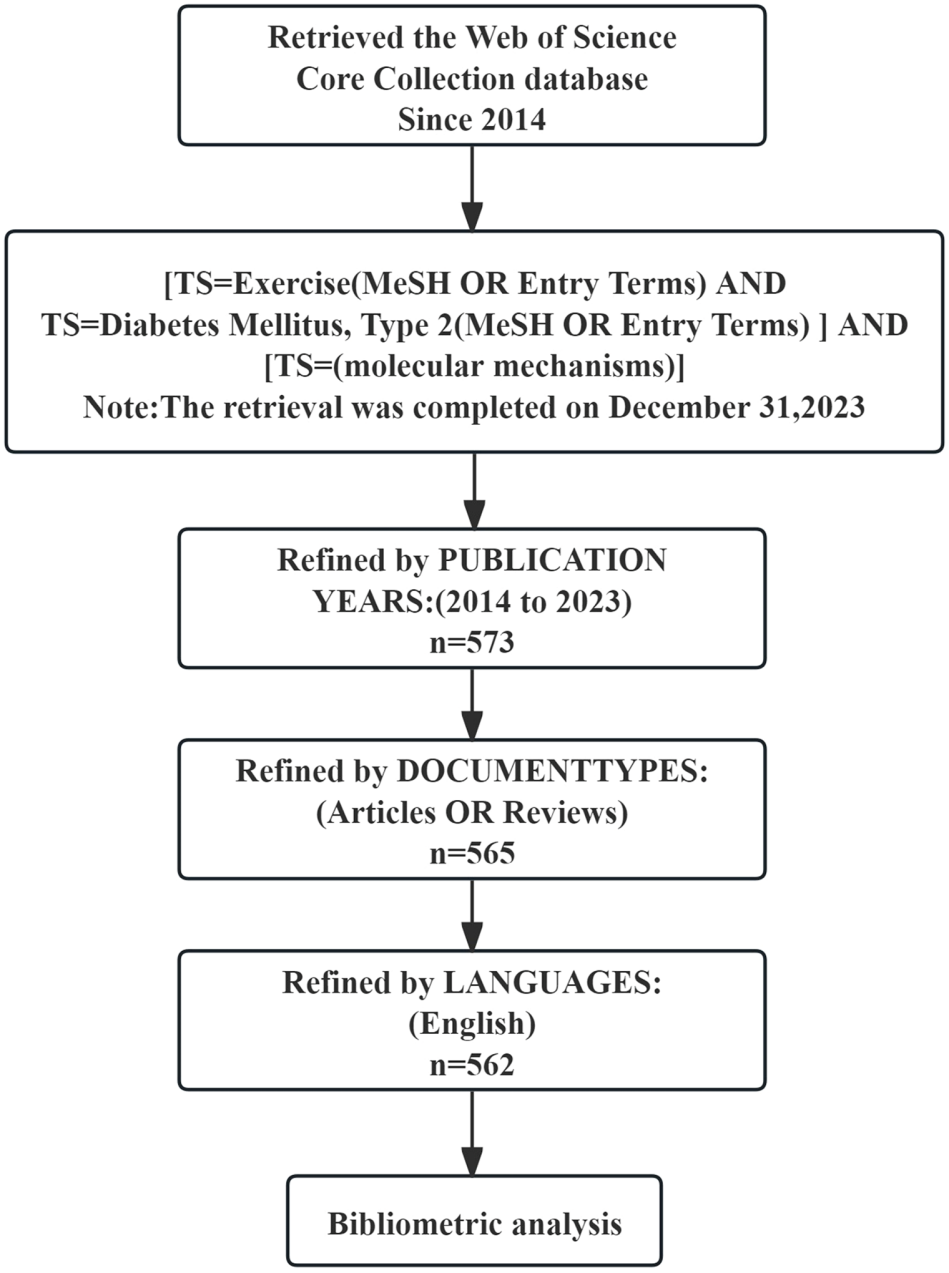
The flowchart illustrates the detailed selection criteria and steps involved in this study’s bibliometric analysis.

Our analysis included peer-reviewed original studies or reviews that discussed the molecular mechanisms underlying exercise’s association with metabolic syndrome. We excluded conference abstracts or corrigendum papers, unpublished articles, repetitive publications, and unconnected articles (**Figure 1**).

### 2.3 Bibliometrics and visualization analysis

We utilized CiteSpace 6.2.R4 to analyze articles in plain text format, containing comprehensive data and references, labeled as “download_XXX.txt” (31). Various techniques in CiteSpace were employed to generate visual knowledge maps, including temporal slicing, thresholding, modeling, pruning, merging, and mapping (32, 33). CiteSpace utilizes fundamental principles such as burst detection, betweenness centrality, and heterogeneous networks to facilitate the identification of prevailing patterns, popular subjects, and cutting-edge areas (34). The maps contain nodes representing authors, institutions, nations, or keywords. The node size reflects the frequency or citation count of each node, while the node color indicates the year of occurrence or citation. Additionally, nodes with purple borders have high betweenness centrality and are often hotspots or turning points in a field.

## 3 Results

### 3.1 Distribution of articles by publications and citations

**Figure 2:**
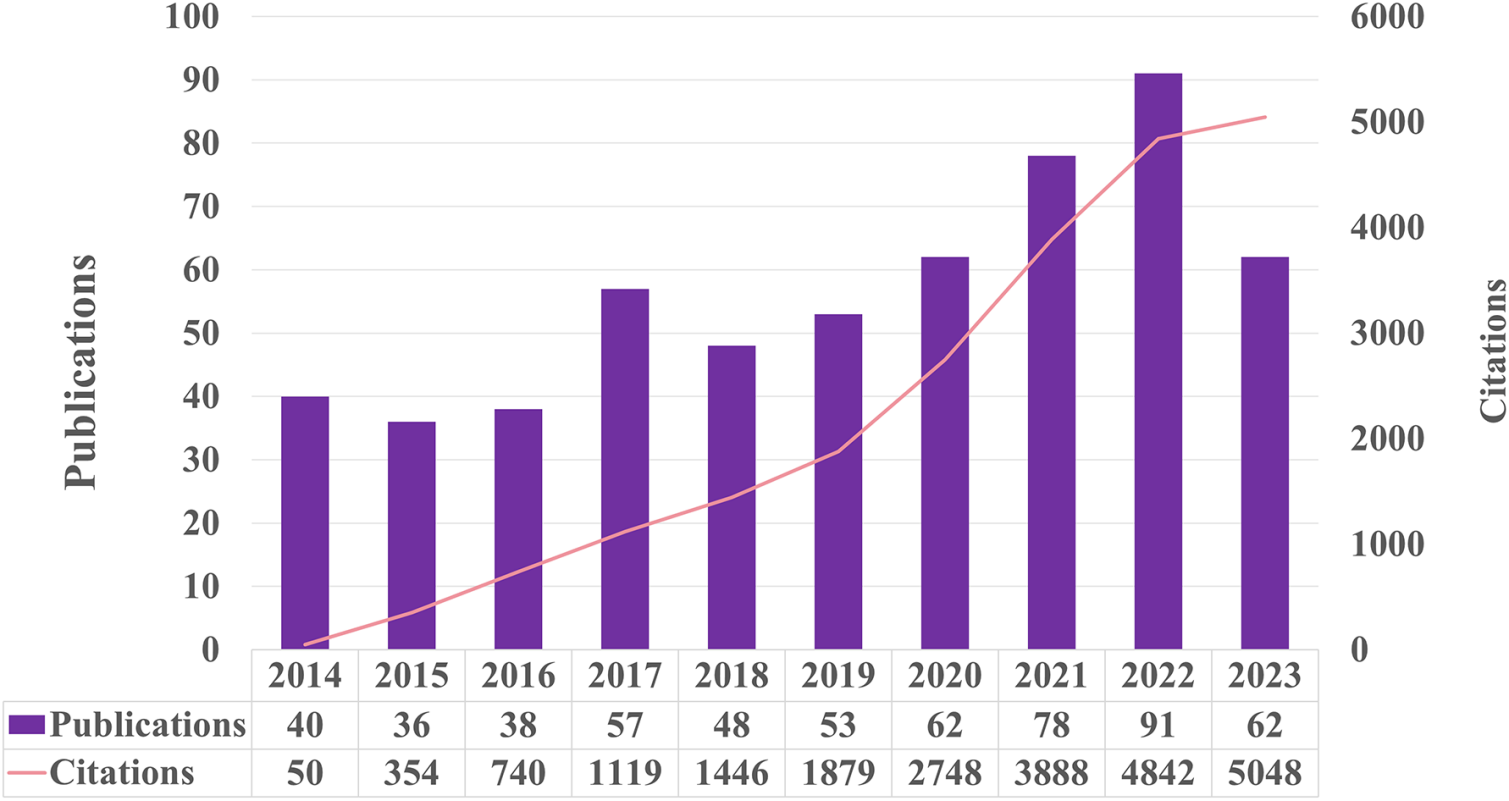
The number of publications and citations each year from 2014 to 2023.

After eliminating records that did not meet the inclusion criteria, a systematic literature search yielded 562 eligible papers. **Figure 2** shows the chronological distribution of the papers from 2014 to 2023. The study investigating the molecular processes of exercise in relation to metabolic syndrome revealed a predominantly positive pattern, albeit with occasional fluctuations. 2022 recorded the peak number of publications, closely followed by 2021 and 2020. However, there was a significant decrease in the number of publications in 2023 compared to 2022. In 2014, the number of publications was at its lowest. The number of citations in this area experienced a significant surge, increasing from 50 in 2014 to 5048 in 2023. Despite a notable decline in the number of publications in 2023, the citation counts in this specific field remained high. The data suggest that although there has been a decrease in the quantity of publications in 2023, this field of study has consistently garnered significant interest over the past decade.

### 3.2 Research cooperation network

#### 3.2.1 Co-authorship

We selected publications using a modified g-index for each one-year time slice, adjusting the number and size of nodes with a scale factor of k = 25 (35, 36). The tree-ring history was applied as the node display pattern to illustrate the evolution of the research field. Collaboration is represented by lines between nodes, with the line color indicating the first year of collaboration.

**Figure 3:**
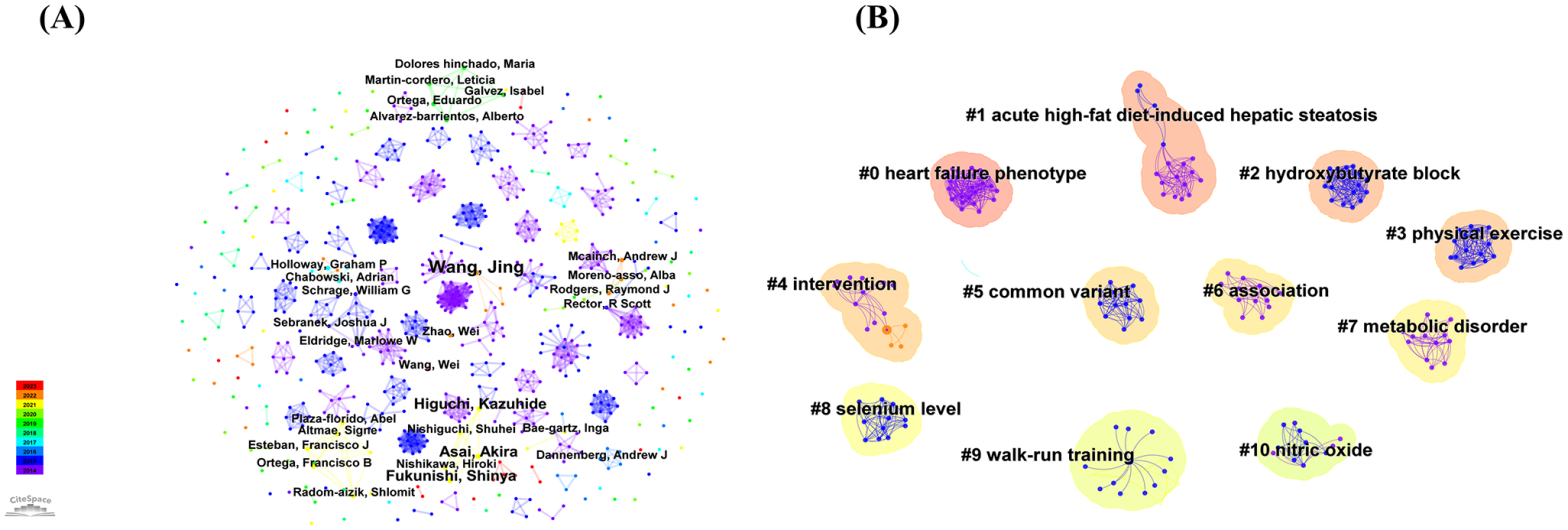
**(A)** The map illustrates the author cooperation network in this field, where the connecting lines signify author interactions. **(B)** The co-authorship network, based on title keywords, reveals distinct clusters labeled 0 to 10, where lower numbers represent larger clusters.

**Figure 3A** shows the important connected part of the merged co-authorship network, which has 689 nodes and 1559 links. It shows which authors worked together most often and how they did it. The co-authorship network showcases highly productive authors and the extent of their collaboration. Despite Wang Jing’s leading output with four publications and the subsequent contributions by Higuchi Kazuhide, Asai Akira, and Fukunishi Shinya, each with three articles, the network revealed a lack of collaboration among authors. Furthermore, the low centrality identified within the network underscores the need for more extensive, high-quality, and large-scale collaborative research initiatives.

The co-authorship network, based on title keywords, reveals several distinct clusters, each corresponding to a specific label. The labels range from 0 to 10, with lower numbers indicating larger clusters (**Figure 3B**). Multiple terms closely related to the titles comprise each cluster. A standard measure for evaluating the quality of clusters is the silhouette value, which indicates how well the terms fit within their assigned cluster (37); a silhouette value above 0.7 suggests high clustering efficiency and validity (38, 39). Using the log-likelihood ratio method, 11 clusters were found with silhouette values above 0.95. These clusters were mostly about acute high-fat diet-induced hepatic steatosis, hydroxybutyrate block, exercise, intervention, common variant, association, metabolic disorder, selenium level, walk-run training, and nitric oxide.

#### 3.2.2 Co-country

**Figure 4:**
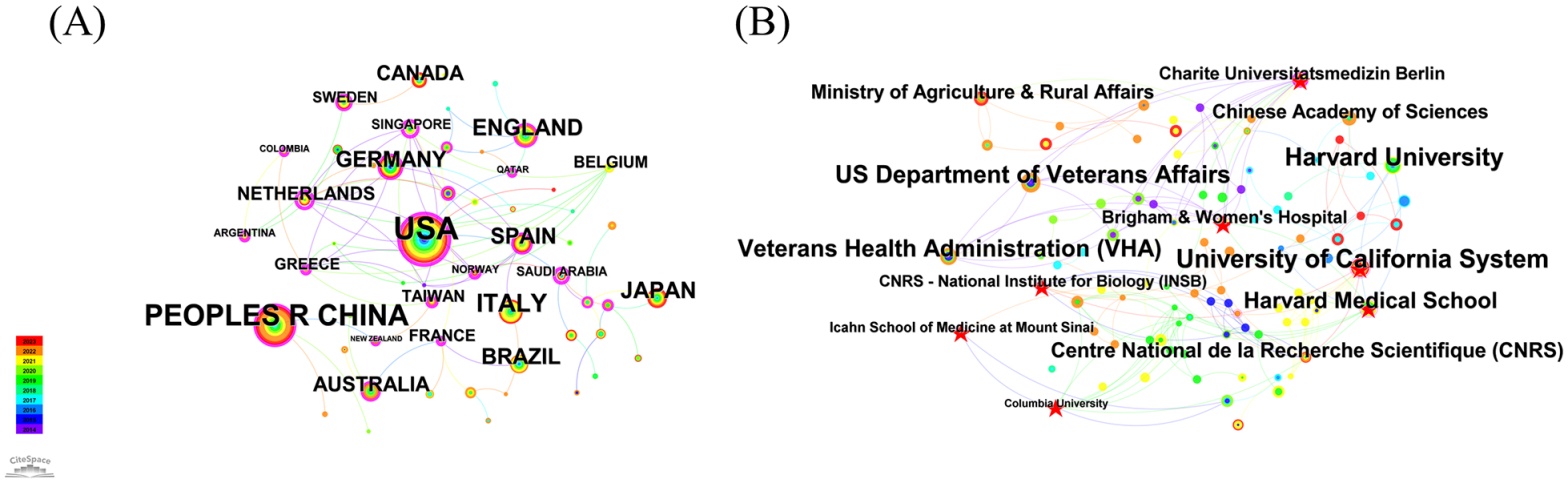
**(A)** The map illustrates the network of country cooperation in this field, where the connecting lines signify the interactions between countries. **(B)** Map of the institute cooperation network in this field, with connecting lines representing institution interactions.

**Figure 4A** presents the co-country network of the merged field, which comprises 59 nodes and 77 links. The most productive country was the USA with 154 articles, followed by China with 111 articles, Italy with 49 articles, England with 35 articles, and Japan with 34 articles. Except for Italy and Japan, the other countries had a betweenness centrality higher than 0.1, indicating their significant role in this research domain.

#### 3.2.3 Co-institution

As shown in **Figure 4B**, the co-institution network map consists of 266 nodes and 396 links. The most productive institutions are the University of California System (13 articles), Harvard University (12 articles), the US Department of Veterans Affairs (11 articles), Veterans Health Administration (VHA) (10 articles), and Harvard Medical School (9 articles). Betweenness centrality measures the importance of each node in a network based on the probability of a random shortest path in the network traversing the node. CiteSpace applies these concepts to identify potential bridges and novel connections in the scientific literature. The University of California System has the highest centrality score, at 0.20. The next one is the Centre National de la Recherche Scientifique (CNRS), which scored 0.12. The third one is the US Department of Veterans Affairs, with a score of 0.08. The fourth one is Charite Universitatsmedizin Berlin, with a score of 0.07. The fifth one is the National Institutes of Health (NIH).

### 3.3 Co-citation

#### 3.3.1 Author co-citation

**Figure 5:**
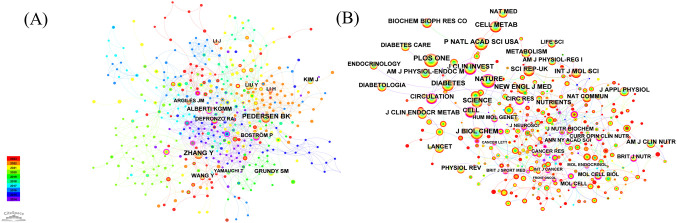
**(A)** Author co-citation analyzes and visualizes author relationships based on their co-citation frequency in academic literature. **(B)** Journal co-citation analyzes and visualizes journal relationships based on their co-citation frequency in academic literature.

**Figure 5A** displays the network of author co-citations. The authors with the highest number of citations were ZHANG Y, receiving 38 citations, followed by PEDERSEN BK with 35, ALBERTI KGMM with 21 citations, and KIM J with 20 citations. Additionally, BOSTRÖM P (0.26), HARDIE DG (0.16), CAI DS (0.15), DEFRONZO RA (0.12), CHEN H (0.12), and CHEN CH (0.12) had high centrality values in the network.

#### 3.3.2 Journal co-citation

The most cited journal in the field was PLOS ONE, with 353 citations, followed by Proceedings of the National Academy of Sciences (284), Nature (276 citations), Diabetes (258 citations), and Journal of Biological Chemistry (241 citations) (**Figure 5B**, **Table 2**). In addition, the journal with the highest centrality score was Cancer Research, with a score of 0.31. The second highest was in the Journal of Neuroscience, with a score of 0.24. The third highest was Cancer Letters, with a score of 0.18. Cell, Current Opinion in Clinical Nutrition and Metabolic Care, Molecular Endocrinology, Annals of the New York Academy of Sciences, and Current Atherosclerosis Reports all had a centrality score of 0.18. The American Journal of Physiology and the International Journal of Cancer both had a centrality score of 0.15.

**Table 2:** The top five cited journal.

Rank	Frequency	Cited journal	IF	Centrality	Cited journal	IF
1	353	Plos One	3.7	0.31	Clin Cancer Res	11.2
2	284	P Natl Acad Sci Usa	11.1	0.24	J Neuro sci	5.3
3	276	Nature	64.8	0.18	Cancer Lett	9.7
4	258	Diabetes	7.7	0.18	Cell	64.5
5	241	J Biol Chem	4.8	0.18	Curr Opin Clin Nutr	3.1

#### 3.3.3 References co-citation

**Table 3** presents a decade of statistical data on co-cited references. The most frequently cited study, Saklayen MG et al.’s 2018 Global Status Report on Metabolic Syndrome (37), provides a detailed analysis of its prevalence, noting its higher incidence in developing countries. Lifestyle modifications, such as regular exercise and caloric restriction, significantly impact management and occurrence. Cruz-Jentoft AJ et al. (2019) (38) highlight how sarcopenia worsens symptoms of metabolic syndrome beyond muscle loss in older adults. Schiattarella GG et al. (2019) (39) found that metabolic syndrome causes inflammation and throws off the balance of nitric oxide, which changes how heart cells respond to stress. Friedman SL et al. (2018) (40) emphasized metabolic syndrome’s role in non-alcoholic fatty liver disease and steatohepatitis, controllable via diet, lifestyle adjustments, and medications. Perakakis et al. (2017) (41) suggested irisin as a promising target for metabolic syndrome intervention. Younossi Z et al. (2018) reviewed non-alcoholic fatty liver disease’s management, underscoring the importance of liver function tests and physical activity (42). Petersen MC et al. (2018)explored insulin resistance processes in muscle, liver, and fat, establishing a comprehensive model (43).

**Table 3:** The top five cited references.

Rank	First author	Cited references	Frequency	Year	Journal
1	Saklayen MG	The Global Epidemicof the Metabolic Syndrome	8	2018	Curr Hypertens Rep
2	Cruz-Jentoft AJ	Sarcopenia: revised European consensus on definition and diagnosis	6	2019	Age Ageing
3	Schiattarella GG	Nitrosative stress drives heart failure with preserved ejection fraction	5	2019	Nature
3	Friedman SL	Mechanisms of NAFLD development and therapeutic strategies	5	2018	Nat Med
3	Perakakis N	Physiology and role of irisin in glucose homeostasis	5	2017	NatRev Endocrinol
3	Younossi Z	Global burden of NAFLD and NASH: trends, predictions, risk factors and prevention	5	2018	Nat Rev Gastro Hepat
3	Petersen MC	Mechanisms of Insulin Action and Insulin Resistance	5	2018	Physiol Rev

**Figure 6:**
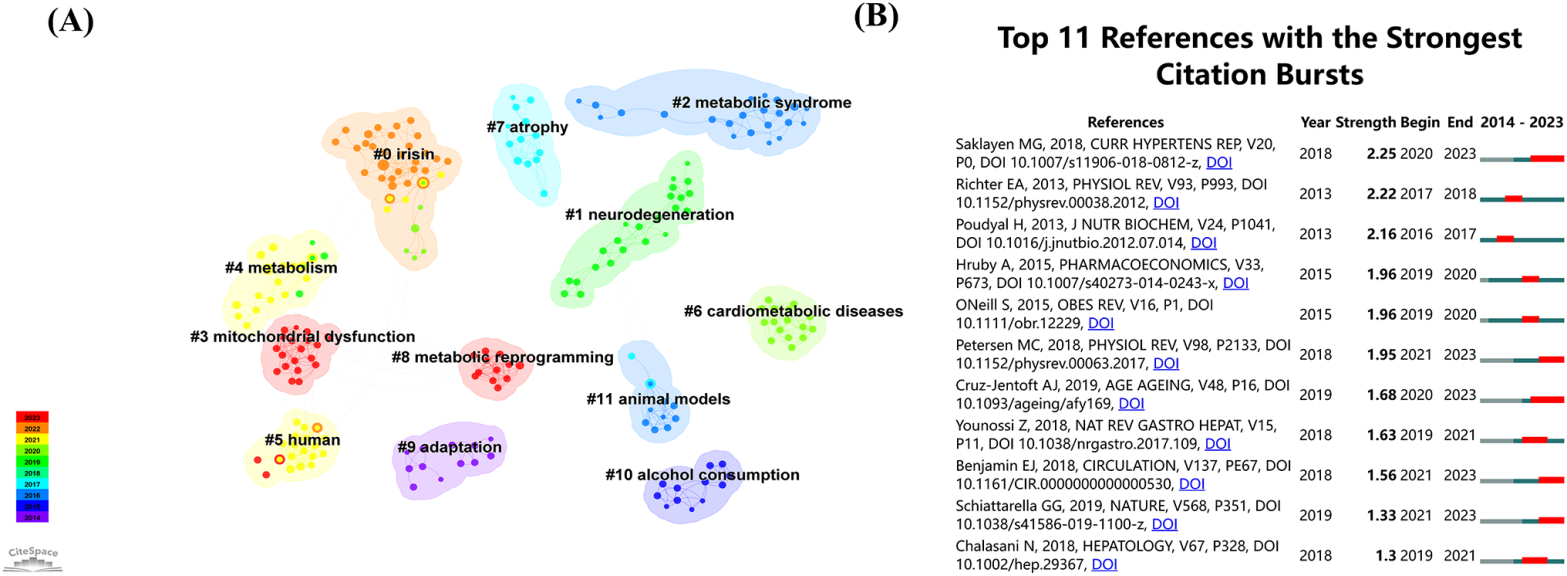
**(A)** The co-references network, based on title keywords, reveals distinct clusters labeled 0 to 10, where lower numbers represent larger clusters. **(B)** The top 11 burst references from 2014 to 2023.

**Figure 6A** displays the 12 most significant clusters of references, frequently cited together, with silhouette values above 0.98. These clusters primarily focus on keywords related to metabolic diseases, such as “irisin,” “neurodegeneration,” “metabolic syndrome,” “mitochondrial dysfunction,” “human,” “cardiometabolic diseases,” “atrophy,” “metabolic reprogramming,” “adaptation,” “alcohol consumption,” and “animal models.” **Figure 6B** highlights the top 11 references with the most intense citation bursts, indicating emerging trends or growing interests in the field. Generally, the most co-cited references also exhibit the most intense citation bursts.

### 3.4 Keyword analysis

#### 3.4.1 Keywords co-citation and clusters

**Figure 7:**
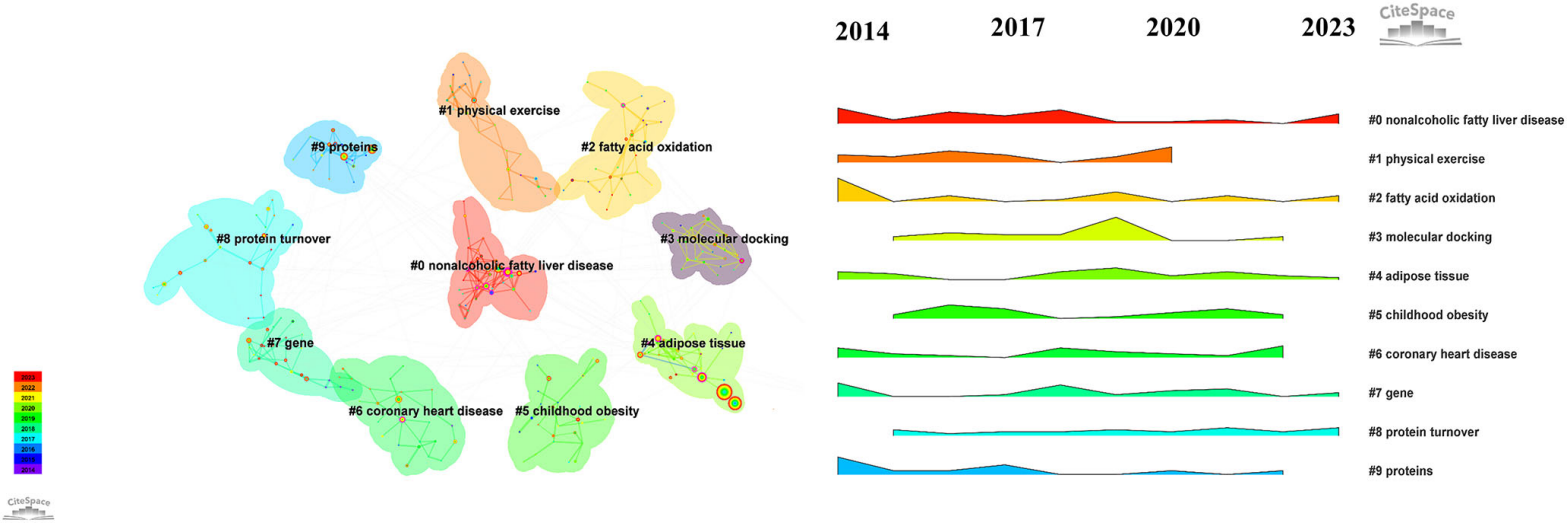
Keyword Cluster reveals distinct clusters labeled 0 to 10, where lower numbers represent larger clusters.

The keywords provide a summary of the main text, highlighting the most relevant and frequent research topics in this field. To identify the keywords, we applied a modified g-index to each annual time slice and selected publications based on a scale factor of k = 25. The resulting network consisted of 358 nodes and 669 links, representing the co-occurrence of keywords. **Table 4** lists the top 10 keywords with high frequency and centrality. Using the loglikelihood ratio method, we successfully generated ten distinct clusters. These clusters covered a range of research areas, including “nonalcoholic fatty liver disease,” “physical activity,” “fatty acid oxidation,” “molecular docking,” “adipose tissue,” “childhood obesity,” “coronary heart disease,” “gene,” “protein turnover,” and “proteins.” Additionally, an analysis of these clusters revealed the ebb and flow of research hotspots over the decade from 2014 to 2023 (**Figure 7**).

**Table 4:** Keyword Frequency and Centrality.

Rank	Frequency	Keywords	Centrality	Keywords
1	173	metabolic syndrome	0.37	expression
2	124	insulin resistance	0.18	stress
3	85	skeletal muscle	0.16	lipid metabolism
4	82	physical activity	0.16	adiponectin
5	73	oxidative stress	0.15	activation
6	65	exercise	0.13	Adipose tissue
7	54	adipose tissue	0.13	diet
8	54	obesity	0.12	risk factors
9	45	expression	0.12	mice
10	41	mechanisms	0.12	Aerobic exercise

#### 3.4.2 Keywords with citation bursts

**Figure 8:**
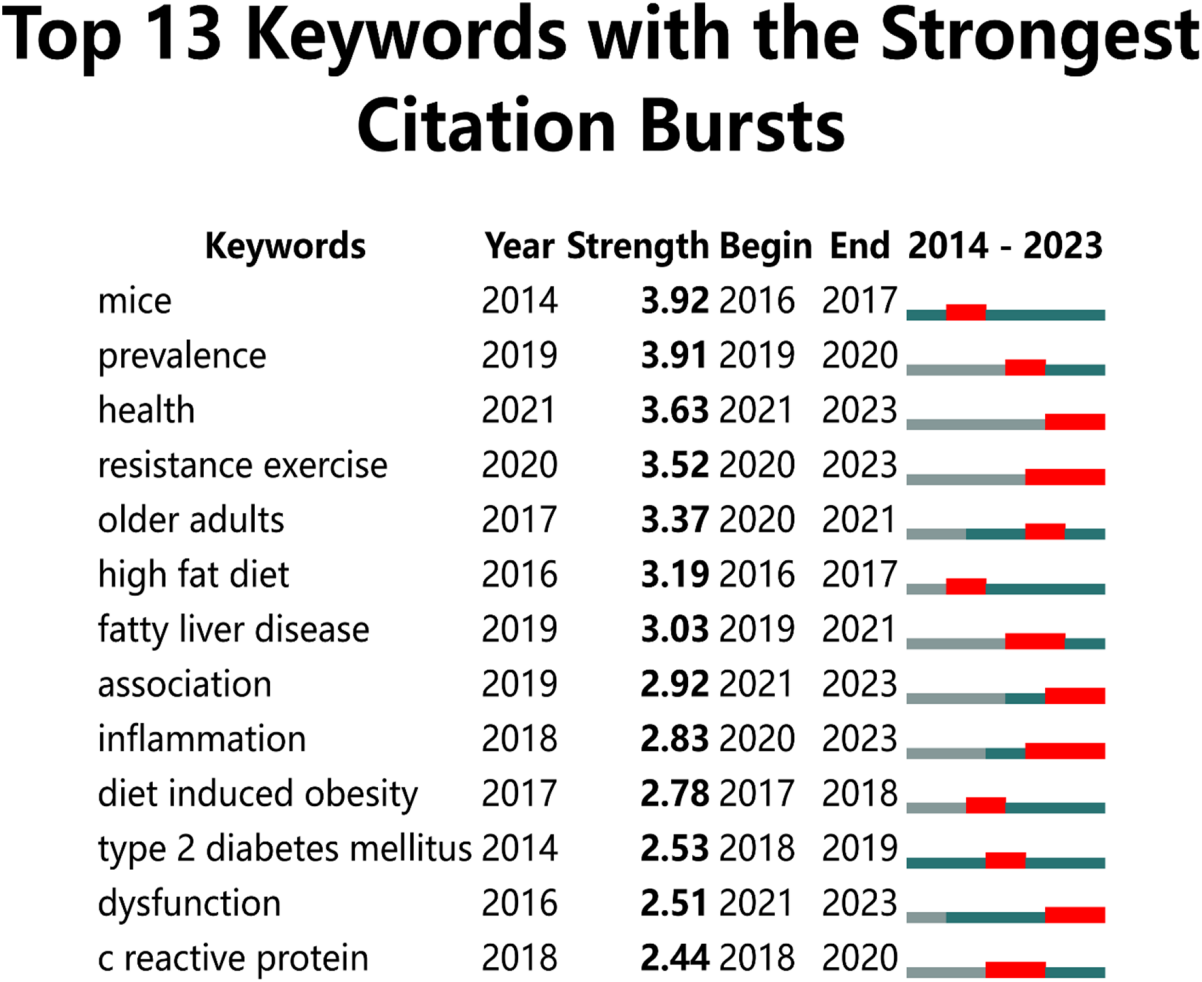
The top 13 burst keywords from 2014 to 2023.

**Figure 8** analyses the top 13 keywords with significant citation bursts in exercise and metabolic syndrome research from 2014 to 2023, highlighting emerging trends. Health-related studies commonly used the leading keyword, “Mice,” with a burst strength of 3.92 during 2016-2017. Following closely, “prevalence” had a burst strength of 3.91 from 2019 to 2020, reflecting a focus on the spread of various health conditions (44–48). **Figure 8** analyses the top 13 keywords with significant citation bursts in exercise and metabolic syndrome research from 2014 to 2023, highlighting emerging trends. Health-related studies commonly used the leading keyword, “Mice,” with a burst strength of 3.92 during 2016-2017. Following closely, “prevalence” had a burst strength of 3.91 from 2019 to 2020, reflecting a focus on the spread of various health conditions (49, 50). Other key terms with significant bursts include “resistance exercise,” “older adults,” “high-fat diet,” “fatty liver disease,” “inflammation,” “diet-induced obesity,” “type 2 diabetes mellitus,” “dysfunction,” and “c-reactive protein,” covering areas like physical fitness, ageing, nutrition, and metabolic diseases.

## 4 Discussion

### 4.1 Trends in the field of research

Based on CiteSpace’s findings, we have summarized the key research trends in the molecular mechanisms of exercise interventions for metabolic syndrome. Recently, an increasing number of researchers, including notable authors like Wang Jing, Higuchi Kazuhide, Asai Akira, and Fukunishi Shinya, have focused on this field. The USA has emerged as a leader, with numerous studies conducted by American universities. The most cited authors and references primarily explore the epidemiology and biochemical signaling of metabolic syndrome. Distinct clusters in the network include heart failure phenotype, acute high-fat diet-induced hepatic steatosis, hydroxybutyrate block, common variant, nitric oxide, irisin, and mitochondrial dysfunction. We note the high frequency and centrality of keywords such as “metabolic syndrome,” “insulin resistance,” “skeletal muscle,” “physical activity,” and “oxidative stress.” The research community has highlighted “resistance exercise,” “health,” “inflammation,” and “dysfunction” as emerging focal points. The keyword “mice” has the highest citation burst, underscoring its prominence in research.

### 4.2 Exercise reverses high-fat diet-induced abdominal obesity

The results of this study show that highly co-cited authors and publications focus on metabolic disorders induced by high-fat diets (**Figures 3**, **6**). High-fat diets easily lead to obesity and non-alcoholic fatty liver disease, which is a core component of metabolic syndrome (51–53). In animal models, high fat intake contributes to the development of metabolic syndrome (54–56). Rodents fed a high-fat diet develop visceral obesity, insulin resistance, hyperlipidemia, endothelial dysfunction, and hypertension (57, 58). Several experimental studies have documented the preventive effect of exercise in animal models before the onset of obesity/metabolic syndrome. Touati et al. divided mice into four groups: a sedentary group, an exercise training group, a diet adjustment group (switching from a high-fat diet to a normal diet), and a group that underwent both exercise training and diet adjustment. The results showed that irrespective of the diet type, exercise training increased the phosphorylation levels of Akt and eNOS (59). This result has been previously reported, indicating that exercise training can achieve these beneficial effects through the shear stress-induced activation of the Akt/eNOS pathway (60). Fan Zheng et al. subjected mice to 12 weeks of swimming training and found that the expression of key metabolic pathway proteins such as PPAR-γ, CPT-1, and MCAD, which were increased by a high-fat diet, was significantly reduced under the influence of exercise. These results suggest that exercise combats high-fat diet-induced metabolic abnormalities by activating the PPAR-γ/CPT-1/MCAD signaling pathway, highlighting the potential value of regular exercise in preventing metabolic syndrome (61).

The myokine exercise-induced irisin has drawn a lot of attention recently in the field of metabolic syndrome prevention and therapy research (**Figure 6A**). Ercan Bastu, MD split female C57BL/6J mice given a high-fat diet into three groups: the control group, the group given an irisin injection, and the group given exercise. The control group gained much more weight than both the exercise group and the irisin group, according to the results. While the exercise group had significantly greater serum irisin levels, the control group also had significantly higher blood glucose and insulin levels. This suggests that exercise can lower obesity, raise serum irisin levels, and enhance insulin sensitivity (62). Furthermore, leptin, a well-known hormonal marker of obesity, is extremely sensitive to variations in energy consumption, especially when there is an energy deficit (63, 64). The study involved 45 men, dividing them into three groups based on their energy consumption: 1,400, 5,000, and 7,000 kcal. The findings demonstrated that only the 5,000 and 7,000 Kcal groups suffered from decreased serum leptin levels. Therefore, the only effective way to reduce blood leptin levels is through long-term endurance exercise that requires a significant energy expenditure (65). In conclusion, a crucial strategy for reversing the metabolic syndrome’s component of abdominal obesity is consistent, moderate-to-high-intensity exercise training.

### 4.3 Impact of exercise on lipid metabolism and inflammation

Metabolic syndrome is characterized by elevated triglycerides, low high-density lipoprotein cholesterol (HDL-C), increased low-density lipoprotein cholesterol (LDL-C), elevated non-high-density lipoprotein cholesterol (Non-HDL-C), elevated total cholesterol levels, and high free fatty acids (66–68). This study’s keywords co-citation and clusters analysis indicates that factors such as beta-hydroxybutyrate (BHB) are important targets for promoting fatty acid oxidation, which can improve dyslipidemia (**Figures 3B**, **7**). Research by Zhou Xu et al. showed that 12 weeks of regular treadmill exercise significantly reduced lipid accumulation and foam cell formation in ApoE/C mice fed a Western diet. This effect was linked to increased serum levels of beta-hydroxybutyrate (BHB). Experimental results demonstrate that BHB treatment, both *in vivo* and *in vitro*, can elevate the protein levels of cholesterol transporters, such as ABCA1, ABCG1, and SR-BI, thereby reducing lipid accumulation (69). Furthermore, research by Melinda E. Tóth found that in hyperlipidemic, high-fat diet (HFD)-fed mice overexpressing apolipoprotein B-100 (APOB-100), serum triglycerides, tumor necrosis factor-alpha (TNF-α) levels, and liver lipid accumulation were significantly higher in male mice compared to female mice. However, a 7-month exercise intervention nearly eliminated liver lipid accumulation in these hyperlipidemic animals, indicating that exercise effectively reduced body weight, serum triglyceride levels, and pro-inflammatory factor expression in the mouse model (70).

Inflammation plays a crucial role in the occurrence and development of metabolic syndrome, which includes a range of conditions such as dyslipidemia, and has become a research hotspot in recent years (**Figure 8**). Tardif Isabelle et al. found that a 12-week aerobic exercise intervention significantly increased HDL-C levels and decreased LDL-C and TG levels (71). These changes correlated with reduced systemic inflammation markers like C-reactive protein (CRP) and tumor necrosis factor-α (TNF-α), indicating that exercise improved lipid metabolism through anti-inflammatory effects (72). Żebrowska Aleksandra et al. studied the anti-inflammatory effects of exercise-induced muscle factors (myokines), such as interleukin-6 (IL-6). They found that moderate exercise increased IL-6 levels, which had anti-inflammatory effects and helped improve lipid profiles. Their blood analyses of athletes showed increased HDL-C and decreased LDL-C and TG levels after exercise (73–75). These studies collectively provide detailed molecular evidence that exercise significantly improves HDL-C, LDL-C, TG, and TC levels in metabolic syndrome patients by inhibiting inflammation, regulating energy and lipid metabolism, and altering gut microbiota.

### 4.4 Exercise-induced mechanisms in insulin sensitivity

There are several different biochemical pathways through which exercise may reverse hyperglycemia caused by the metabolic syndrome (76–78). The combined impacts of the terms “aerobic exercise” and “resistance exercise” have garnered more international scholarly interest in recent years, according to the bibliometric results of this study (**Figures 6A**, **8**). They describe how exercise improves insulin sensitivity and reverses the molecular processes of hyperglycemia by strengthening the insulin signaling pathway, activating the AMP-activated protein kinase (AMPK) pathway, and having anti-inflammatory effects. Insulin resistance is the main pathophysiological mechanism underlying metabolic syndrome (MS) (43, 79, 80). Khoa Do and colleagues have examined the early metabolic alterations in the triple transgenic Alzheimer’s disease mouse model and their connection to the hypothalamus. Eight weeks of voluntary exercise training reduced apoptosis and increased the number of neurons expressing POMC and NPY in the hypothalamus, improving insulin sensitivity. Researchers discovered that four weeks of voluntary exercise training was enough to reverse the gene expression of inflammatory and apoptotic markers in the hypothalamus; six weeks of exercise improved glucose metabolism; and all three groups improved insulin sensitivity (81).

The notable increase in muscle 5’ AMP-activated protein kinase (AMPK) and its association with metabolic syndrome have attracted much attention in current medical research (82–84). Mark A. South and his colleagues conducted research to investigate the effects of resistance training on individuals diagnosed with metabolic syndrome (MS). Throughout an eight-week resistance training program, every participant experienced enhancement in both maximum strength and endurance. In addition, there was an increase in muscle 5’ AMP-activated protein kinase. It regulates energy metabolism by monitoring fluctuations in the intracellular ratio of AMP to ATP, thereby providing defense against various metabolic stressors (85). In addition, aerobic exercise (AE) enhances insulin sensitivity through many methods (86–89). For example, in individuals with metabolic syndrome (MS), it increases AMP levels by decreasing mTOR concentrations (82). Additional routes include increasing insulin transport protein production and mitigating insulin resistance mechanisms (90, 91). As a cellular energy detector, AMPK enhances glucose absorption and facilitates the transfer of GLUT4 to the outer membrane of cells during muscle contractions and energy depletion (83, 92). Both resistance and aerobic activities improve muscle glucose uptake during and after exercise, hence regulating metabolic equilibrium independently of insulin (93, 94).

### 4.5 Cardiovascular adaptation to exercise-induced stress

Improving heart and vascular function is crucial for preventing metabolic syndrome due to the interactions between cardiovascular function and factors like insulin resistance, central obesity, and dyslipidemia (95, 96). This study, illustrated in **Figures 3B** and **6A**, highlights significant clusters such as heart failure phenotype, cardiometabolic diseases, and nitric oxide. Exercise-induced cardiac expansion is closely linked to enhanced heart function, angiogenesis, cardiomyocyte renewal, and activation of cardiac stem cells (hCSCs) (97). Adult rats that performed intensity-controlled treadmill exercise showed improved heart function and increased myocardial mass due to hCSCs activation, cardiomyocyte hypertrophy, neo-cardiomyocyte generation, and capillary formation (98).Clinical studies have found that the heart releases factors like natriuretic peptide in response to stress, inducing lipolysis in human adipocytes and raising plasma non-esterified fatty acids, linking mechanical forces to endothelin (ET) metabolism (99). Patients with obesity and metabolic syndrome have lower plasma natriuretic peptide levels (100). In athletes, endurance exercise elevates heart IGF-1 expression and activates the PI3K pathway, promoting cardiomyocyte hypertrophy (101). Overexpression of the IGF-1 receptor (IGF-1R) leads to larger cardiomyocytes without cell death or disintegration, enhancing contraction ability (102).

Exercise has been shown to mediate nitric oxide (NO) production (**Figure 3B**), which plays a crucial role in mitigating metabolic syndrome through various molecular mechanisms (103, 104). NO is produced by endothelial cells and is pivotal in promoting smooth muscle relaxation and vasodilation, as well as possessing antithrombotic and anti-atherosclerotic properties (99). The release of NO during exercise significantly reduces cardiovascular risk, a benefit not entirely explained by traditional risk factor adjustments (105, 106). One of the key molecular regulators in this process is Kruppel-like factor 2 (KLF2), which connects mechanical stimuli from exercise to the production of NO synthases in endothelial cells (107). This mechanosensory input ensures that NO synthase levels are appropriately elevated in response to physical activity (108). Moreover, NO not only induces vasodilation and exerts antithrombotic effects but also activates protective enzymes like SIRT1 (109, 110). The presence of shear stress during physical activity is a significant factor in the increased production of NO, which in turn offers protective effects against atherosclerosis, a common complication in individuals with metabolic syndrome (111).

## 5 Conclusions

**Figure 9:**
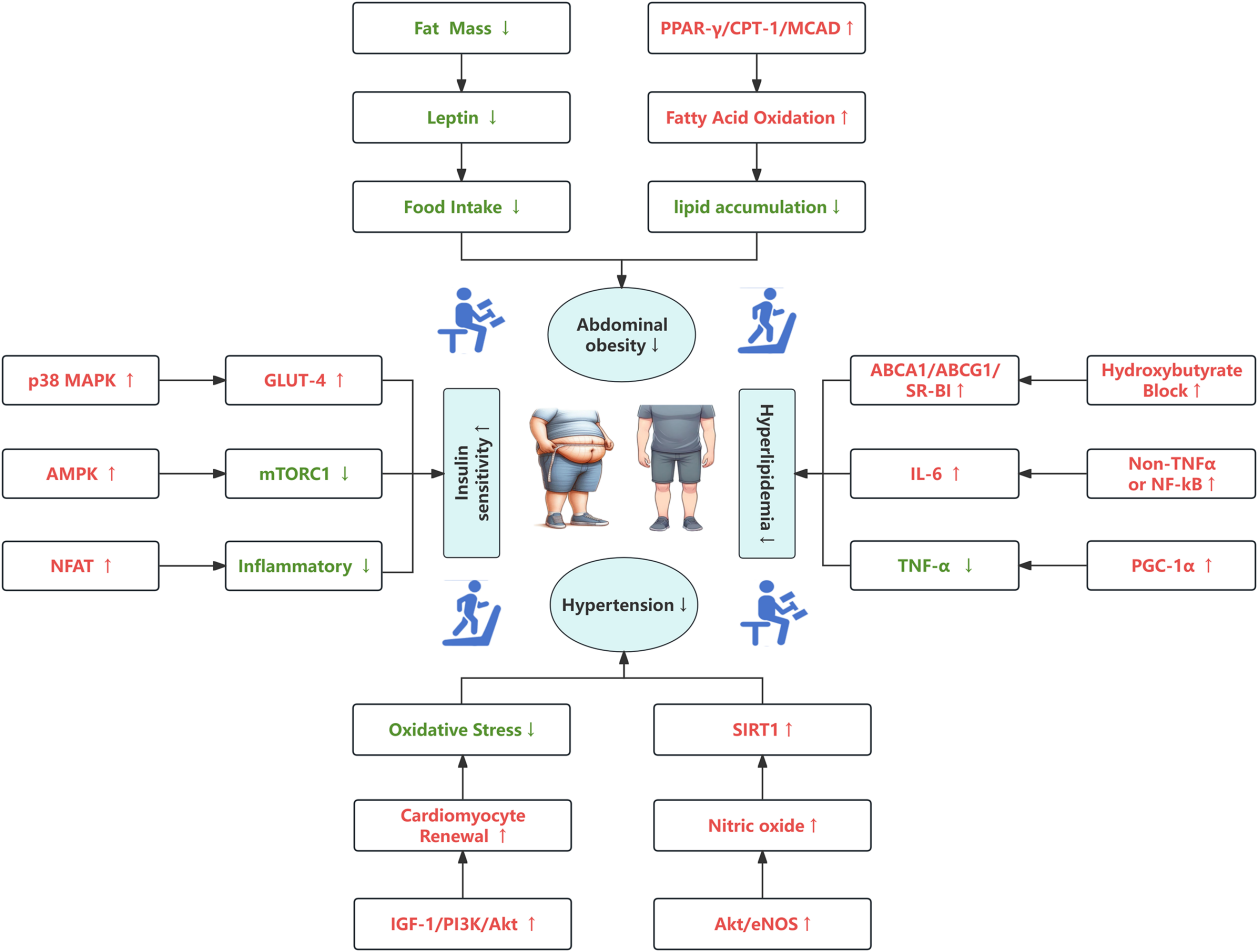
A schematic of the molecular mechanism of exercise training, with red fonts and arrows representing “activation” or “improvement” and green fonts and arrows representing “inhibition” or “reduction”.

This study provides an in-depth analysis of the molecular mechanisms through which exercise influences metabolic syndrome, leveraging bibliometrics and visualization techniques to uncover key research trends and collaborative networks. Our findings highlight the significant role of exercise in mitigating metabolic syndrome through various pathways, including the reversal of high-fat diet-induced abdominal obesity, reduction of lipid accumulation and inflammation, enhancement of insulin sensitivity, and improvement of cardiovascular function. Our research underscores the importance of regular, moderate-to-high-intensity exercise as a critical strategy for managing metabolic syndrome. Exercise effectively combats metabolic abnormalities by activating pathways such as the PPAR-γ/CPT-1/MCAD signaling, increasing the levels of beneficial myokines like irisin, and improving insulin sensitivity through the activation of AMPK and other insulin signaling pathways. Moreover, exercise-induced nitric oxide production plays a pivotal role in promoting cardiovascular health by enhancing vasodilation, reducing atherosclerosis, and activating protective enzymes like SIRT1(**Figure 9**).

The bibliometric analysis reveals that the USA leads in research output and citations, with prominent contributions from American universities and researchers. Despite a decline in publications in 2023, the field has consistently garnered significant interest over the past decade. Our study also highlights the need for more extensive and high-quality collaborative research initiatives to advance our understanding of exercise’s molecular impacts on metabolic syndrome. In conclusion, our comprehensive analysis provides valuable insights into the molecular mechanisms by which exercise ameliorates metabolic syndrome. This knowledge is crucial for researchers and policymakers in developing effective strategies for preventing and managing this complex condition. Future research should focus on expanding collaborative efforts and exploring novel molecular targets to further enhance the therapeutic potential of exercise in metabolic health.
